# OSlgg: An Online Prognostic Biomarker Analysis Tool for Low-Grade Glioma

**DOI:** 10.3389/fonc.2020.01097

**Published:** 2020-07-07

**Authors:** Yang An, Qiang Wang, Lu Zhang, Fengjie Sun, Guosen Zhang, Huan Dong, Yingkun Li, Yanyu Peng, Haojie Li, Wan Zhu, Shaoping Ji, Yunlong Wang, Xiangqian Guo

**Affiliations:** ^1^Department of Predictive Medicine, Institute of Biomedical Informatics, Cell Signal Transduction Laboratory, Bioinformatics Center, Henan Provincial Engineering Center for Tumor Molecular Medicine, Kaifeng Key Laboratory of Cell Signal Transduction, School of Basic Medical Sciences, School of Software, Henan University, Kaifeng, China; ^2^Department of Anesthesia, Stanford University, Stanford, CA, United States; ^3^Henan Bioengineering Research Center, Zhengzhou, China

**Keywords:** low-grade glioma, prognostic biomarker, gene expression profiling, survival analysis, survival outcome

## Abstract

Glioma is the most frequent primary brain tumor that causes high mortality and morbidity with poor prognosis. There are four grades of gliomas, I to IV, among which grade II and III are low-grade glioma (LGG). Although less aggressive, LGG almost universally progresses to high-grade glioma and eventual causes death if lacking of intervention. Current LGG treatment mainly depends on surgical resection followed by radiotherapy and chemotherapy, but the survival rates of LGG patients are low. Therefore, it is necessary to use prognostic biomarkers to classify patients into subgroups with different risks and guide clinical managements. Using gene expression profiling and long-term follow-up data, we established an **O**nline consensus **S**urvival analysis tool for LGG named OSlgg. OSlgg is comprised of 720 LGG cases from two independent cohorts. To evaluate the prognostic potency of genes, OSlgg employs the Kaplan-Meier plot with hazard ratio and p value to assess the prognostic significance of genes of interest. The reliability of OSlgg was verified by analyzing 86 previously published prognostic biomarkers of LGG. Using OSlgg, we discovered two novel potential prognostic biomarkers (*CD302* and *FABP5*) of LGG, and patients with the elevated expression of either *CD302* or *FABP5* present the unfavorable survival outcome. These two genes may be novel risk predictors for LGG patients after further validation. OSlgg is public and free to the users at http://bioinfo.henu.edu.cn/LGG/LGGList.jsp.

## Introduction

Glioma is the most frequent primary brain tumor with four grades from grade I to IV. Grade IV glioma is also known as glioblastoma, while grade II and III glioma refer to low-grade glioma (LGG) designated by World Health Organization (WHO) (1–4). LGG includes three histological types: astrocytoma, oligodendroglioma, and oligoastrocytoma (4–6), while oligoastrocytoma is no longer considered as a separate entity since the current WHO classification has included molecular markers (including IDH1 mutation and 1p/19q codeletion) to identify astrocytoma and oligodendroglioma, not oligoastrocytoma (3, 7). Although less aggressive than high-grade glioma, LGG eventually advances to high-grade glioma without intervention therapy (5, 8). For most LGG patients, the treatment is surgical excision followed by radiotherapy and/or chemotherapy including temozolamide (TMZ) and PCV (combination of procarbazine, lomustine, and vincristine) (5, 9). However, some patients would be tolerant or resistant to such uniform treatment and progress to relapse and eventual lead to death faster than the others (5, 8), maybe due to the molecular heterogeneity of LGG (10–12), so the optimum timing of the therapeutic schedule needs to be determined case by case (13).

With the availability of public gene expression profiling data, more and more molecular predictive and prognostic indicators have recently been identified in LGG to guide the personalized therapy by informing which patients require early intervention and predicting the prognosis outcome (6, 14). However, it requires specific bioinformatics skills to perform prognosis analysis using these gene expression profiling data. It is desirable that users with limited bioinformatics skills can assess prognostic biomarkers for LGG using a convenient and easy-to-use bioinformatics tool. In the present study, we developed an easy-to-use web server named OSlgg, which provides a platform to evaluate the prognostic value of a gene of interest by applying Kaplan-Meier plot to present the association between candidate gene and survival rate, conduce to the clinical translation of potential prognostic biomarkers and targeted therapies for LGG patients.

## Methods

### Data Collection

Gene expression profiling and related long-term follow-up data of low-grade gliomas were collected from GEO (Gene Expression Omnibus) and TCGA (The Cancer Genome Atlas) database. For dataset searching, the keywords, including “low-grade glioma,” “gene expression,” and “survival” were used in GEO database. The criteria for dataset accession are as followed: (1) has gene expression profiling data; (2) includes the long-term follow-up data of patients; (3) contains more than 50 LGG cases to enable valid survival analysis. Thus, one GEO dataset (GSE107850) with 195 LGG cases was collected (Table 1). For TCGA dataset, gene expression profiling (RNA-seq, level-3, HiSeqV2) and follow-up data of 525 LGG cases were downloaded in 2019 (Table 1). The survival terms of follow-up data include OS (overall survival), RFS (relapse-free survival) and PFS (progression-free survival) (Table 1). And the clinicopathologic characteristics of LGG patients are summarized in Table S1.

**Table 1:** Summary of datasets in OSlgg.

**Dataset**	**Sample size**	**Data type**	**Platform**	**Survival terms**
TCGA	525	RNA-seq	Illumina HiSeqV2	OS, RFS
GSE107850	195	cDNA array	GPL14951	PFS
Total	720			

### Development of OSlgg

**Figure 1:**
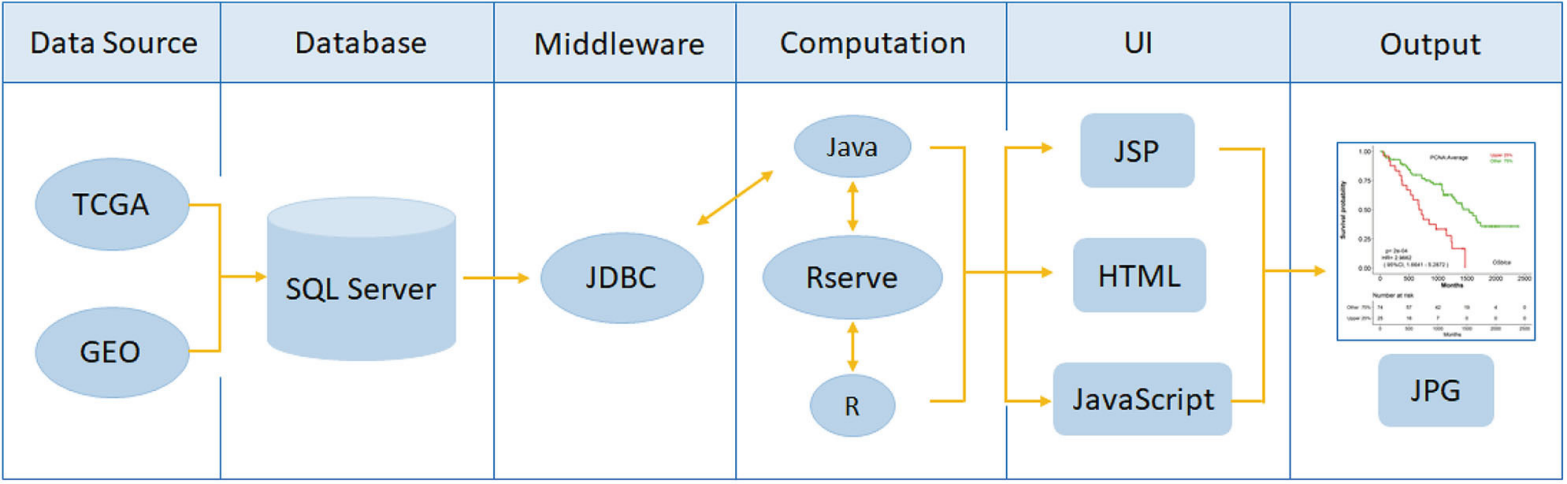
Flowchart of web server OSlgg architecture.

OSlgg adopts object-orient programming method to develop each function module based on the structure of B/S (Browser/Server). Java and R are used to achieve server-side. The web server function was divided into three parts, including UI (user interface), data analysis and data access. Java and R language are used for data analysis and data access, respectively. UI is developed by HTML5, JQurey, and JSP. And the real time communication between web server and clients is achieved by Servlet. Gene expression profiling and clinical data were stored in relational tables in SQL Server database. System architecture flow diagram is presented in Figure 1, as previously described (15–18). OSlgg can be accessed at bioinfo.henu.edu.cn/LGG/LGGList.jsp.

### Verification of Prognostic Biomarkers in OSlgg

To assess the reliability of prognostic analysis of OSlgg web server, previously published prognostic biomarkers of LGG were searched in PubMed using the keywords “low-grade glioma,” “survival,” “prognosis” and “biomarker.” As a result, we collected 93 papers with 86 reported prognostic biomarkers. The prognostic abilities of these prognostic genes were assessed in OSlgg.

### Discovery of Novel Prognostic Biomarkers in OSlgg

To identify novel prognostic biomarker for LGG, we genome-widely analyzed the prognostic values of human genes using Cox regression analysis. Genes significantly related to prognosis were selected (cox *p* value < 0.05), including *CD302* and *FABP5*. As they exhibited significant correlation with prognosis (*p* value < 0.000001) in Cox regression analysis, we further evaluated the prognostic values of *CD302* or *FABP5* in OSlgg. In addition, correlation analysis and GSEA (Gene Set Enrichment Analysis) were performed to investigate the functions of *CD302* and *FABP5*. Correlations between the expression levels of *CD302* or *FABP5* and 86 previously reported LGG prognostic biomarkers were assessed using Spearman's rank correlation test of a non-normal distribution as continuous measures and TCGA data. For GSEA analysis, patients from TCGA cohort were split into two subgroups according to *CD302* or *FABP5* expression, named as CD302 or FABP5 Upper 25% expression and Lower 75% expression. Then GSEA was run to investigate the gene sets enriched in each subgroup.

### Statistical Analysis

Statistical evaluation was performed with SPSS 19.0 (SPSS Inc., Chicago, IL, USA) and GraphPad Prism 7.0 (GraphPad Inc., La Jolla, CA, USA). The association between *CD302/FABP5* expression and clinicopathological characteristics was measured by using Chi-square test. Students' t-test and one-way ANOVA (analysis of variance) were employed to determine the significance of expression difference of *CD302/FABP5* expression in distinct histologic grades and primary therapy outcomes, respectively. Univariate and multivariate cox regression analysis of *CD302/FABP5* expression and clinical factors associated with survival of LGG patients were conducted by using SPSS. A value of *p* < 0.05 was considered to be statistical significant.

## Results

### Clinical Features of LGG Patients in OSlgg

**Figure 2:**
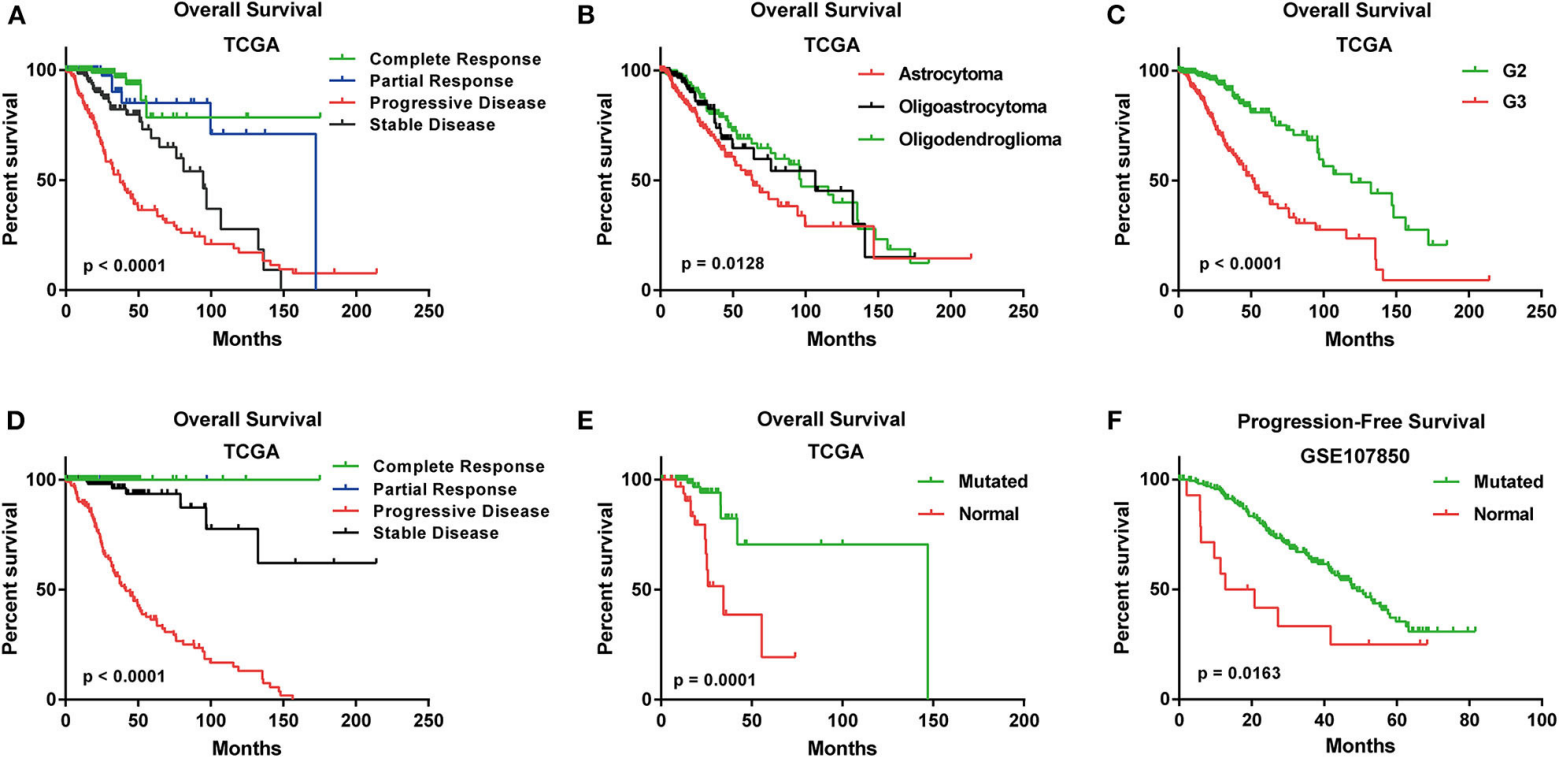
Survival analysis of LGG patients with different clinical features in OSlgg. Kaplan-Meier plots for **(A)** Primary therapy outcome, **(B)** Histological type, **(C)** Neoplasm histologic grade, **(D)** Follow-up therapy outcome, **(E)** IDH status in terms of OS in TCGA cohort; **(F)** IDH status in terms of PFS in GSE107850 cohort.

In TCGA cohort, the median age of 525 LGG patients is 41. Three histological types were included. Specifically, astrocytoma accounts for 37% of all the LGG patients (*n* = 196), oligoastrocytoma accounts for 26% (*n* = 134) and oligodendroglioma accounts for 37% (*n* = 195) (Table S1). A summary of clinical features for each cohort was shown in Table S1. The Kaplan-Meier plots for LGG patients in OSlgg grouped by different histological type, histologic grade, IDH status, primary and follow-up therapy outcome were presented in Figure 2. As shown, these clinical features were significantly associated with survival (OS or PFS), respectively (Figure 2).

### Application of OSlgg

**Figure 3:**
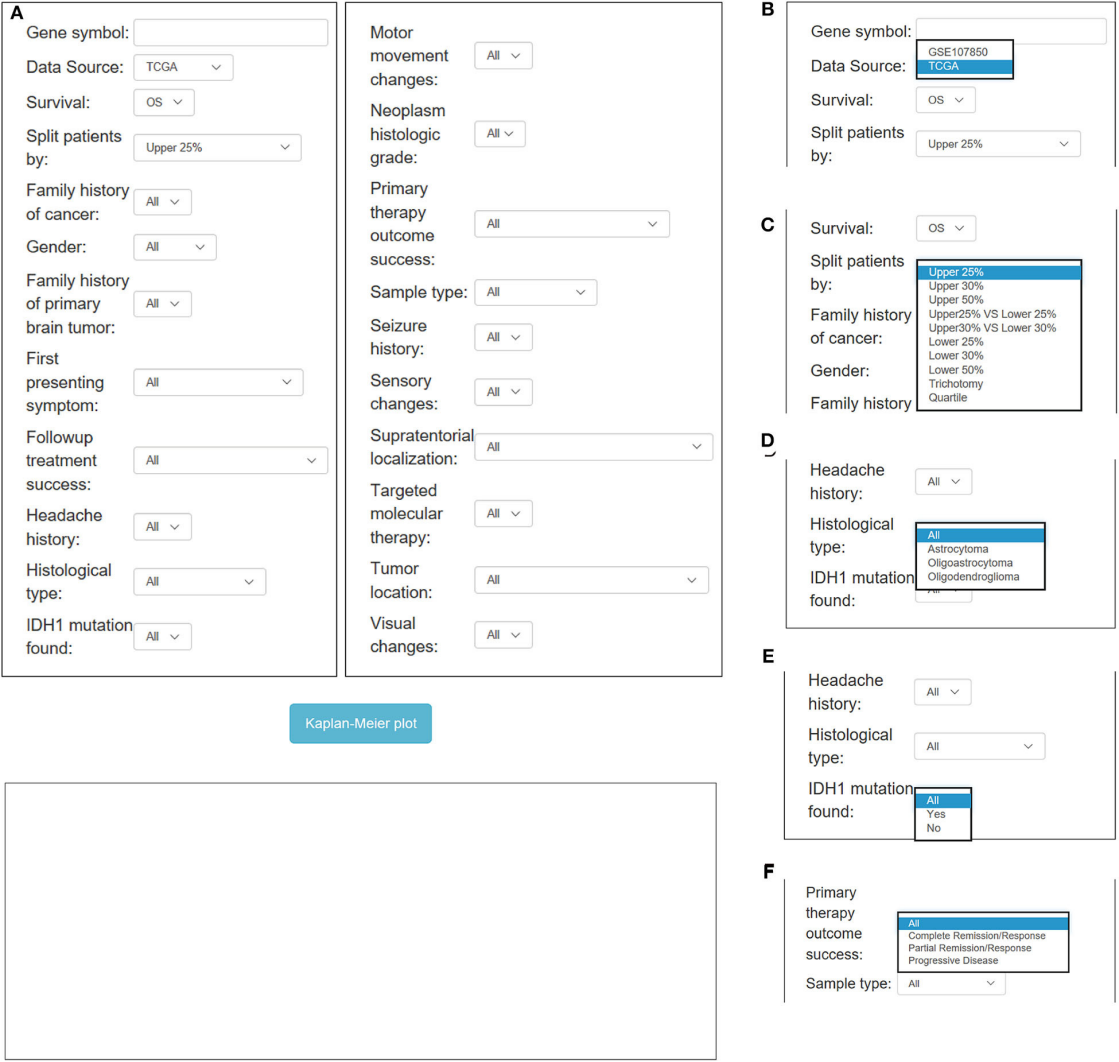
Overview of OSlgg subfield interface for TCGA cohort. **(A)** Screenshot of OSlgg main interface. **(B–F)** Input interfaces of OSlgg for Data source **(B)**, cut-off **(C)**, Histological type **(D)**, IDH1 mutation **(E)**, and therapy outcome **(F)**.

**Figure 4:**
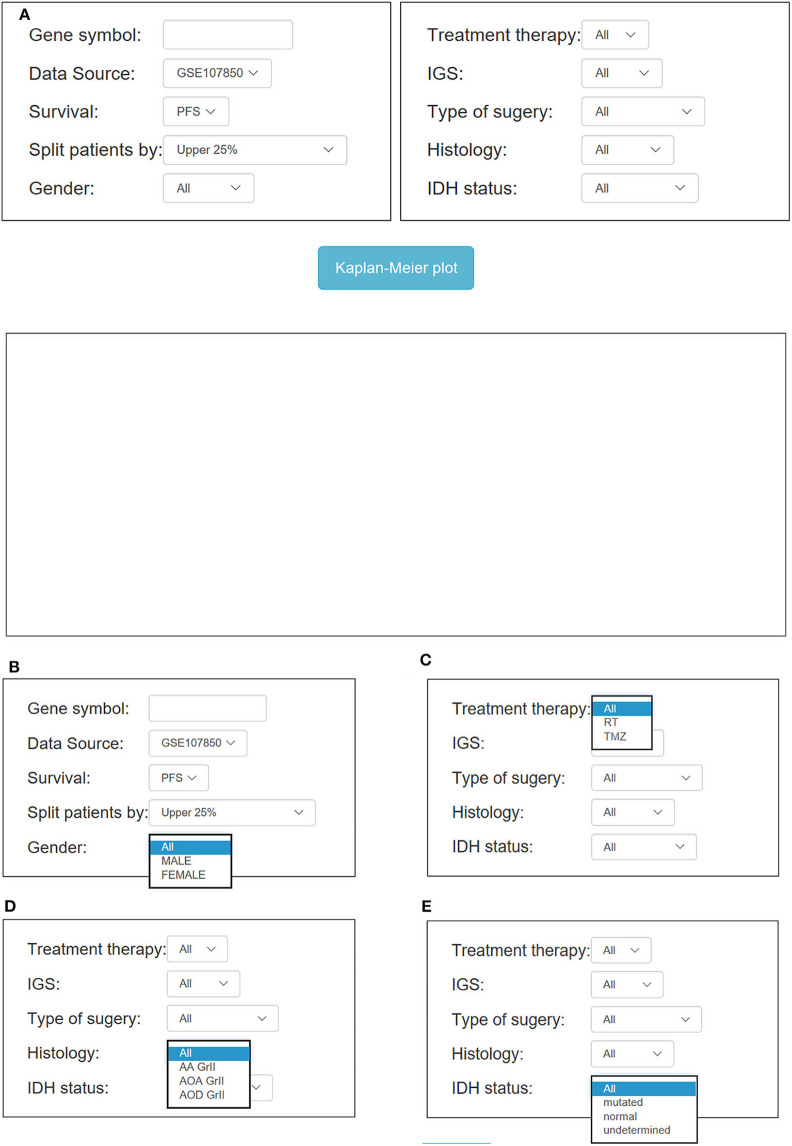
Overview of OSlgg subfield interface for GSE107850 cohort. **(A)** Screenshot of OSlgg main interface. **(B–E)** Input interfaces of OSlgg for Gender **(B)**, Treatment **(C)**, Histology **(D)**, and IDH status **(E)**.

In OSlgg, “Gene symbol,” “Data Source,” “Survival,” and “Split patients by” are set as the four main parameters to assess the prognostic value of a gene of interest (Figures 3, 4). Typically, the official gene symbol is required to be filled into the “Gene symbol” input box by users. Drop-down menu of “Data source” offers two options for users to pick either of the two independent cohorts (TCGA and GSE107850) (Figure 3B). Next, users may select the cut-off, by which patients can be split into 2-4 groups according to the expression of the inquired gene (Figure 3C). Furthermore, according to user's special needs, users may divide LGG patients into subgroups by setting different clinical factors, such as histological type, IDH status, therapy outcome, gender, treatment, etc. (Figures 3, 4). Then user could click the “Kaplan-Meier plot” button, OSlgg will receive the query and output the analysis results to users in a graphical manner on the web page, present the Kaplan-Meier survival curve, HR (with 95% confidence interval) and *p* value.

### Verification of Previously Published LGG Prognostic Biomarkers in OSlgg

**Figure 5:**
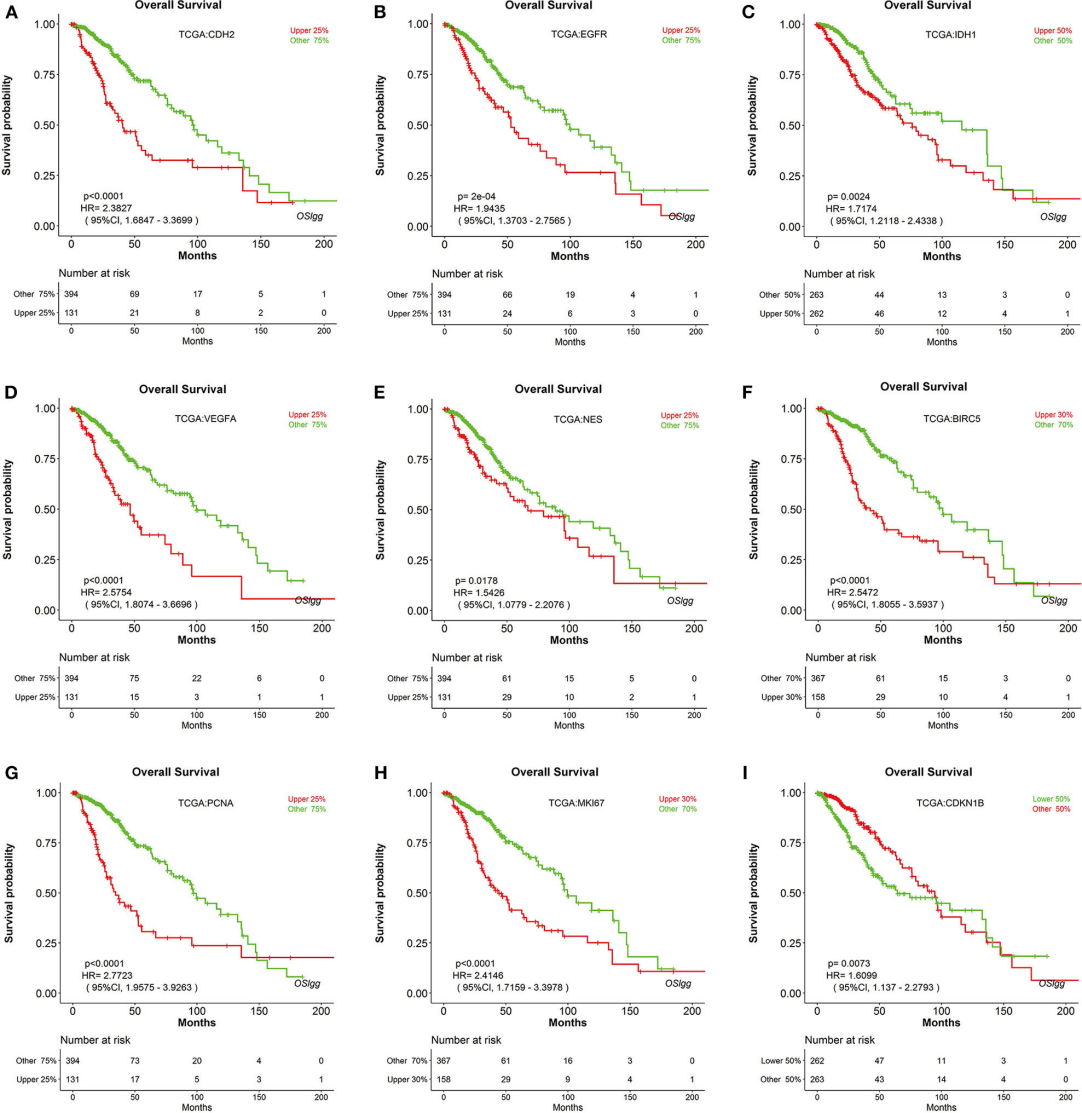
Verification of previously reported prognostic biomarkers in OSlgg. Kaplan[[Inline Image]]-Meier plots for **(A)** *CDH2*, **(B)** *EGFR*, **(C)** *IDH1*, **(D)** *VEGFA*, **(E)** *NES*, **(F)** *BIRC5*, **(G)** *PCNA*, **(H)** *MKI67*, and **(I)** *CDKN1B* in terms of OS. *p*-value, confidence interval (95%CI) and number at risk are as shown. The y-axis represents survival rate and the x-axis represents survival time (months).

To test the reliability of OSlgg web server in prognosis analysis, we collected 86 previously published prognostic biomarkers from 93 papers, including β-catenin, NF-kB, vimentin, Cyclin A, CD31, etc., and assessed their prognostic performances in OSlgg. The analysis results by OSlgg showed that all the 86 biomarkers have predictive values in OSlgg, which was consistent with previous reports (Table 2 and Table S2, Figure 5 and Figure S1), and the housekeeping genes were also presented as negative controls (Figure S2). Among these, N-cadherin (encoded by *CDH2* gene), EGFR, IDH1, VEGF, nestin (encoded by *NES* gene), survivin (encoded by *BIRC5* gene), PCNA, Ki-67 (encoded by *MKI67* gene), and p27 (encoded by *CDKN1B* gene) were frequently reported as risk predictors for LGG (19–34). As previously described, these genes were significantly associated with survival (OS, RFS and PFS) in OSlgg (Table 2, Figure 5 and Figure S1). The elevated expression of *CDH2, EGFR, IDH1, VEGFA, NES, BIRC5, PCNA*, and *MKI67* indicated the unfavorable outcome, while the increased *CDKN1B* expression predicted a favorable outcome for LGG patients (Table 2, Figure 5 and Figure S1). In the remaining 77 biomarkers, 59 genes were adverse predictors, and 18 genes were beneficial predictors (Table S2).

**Table 2:** Verification of previously reported LGG prognostic predictors in OSlgg.

**Gene symbol**	**Biomarker name**	**Clinical survival terms**	**In OSlgg**					**In reference**					**Worse prognosis (expression)**	**References**
			**Cut-off**	***p* value**	**HR**	**95%CI**	**Case**	**Cut-off**	***p* value**	**Case**	**Detection level**	**Validation**		
CDH2	N-cadherin	OS	Upper 25%	<0.0001	2.3827	1.6847-3.3699	525		OS: *p* < 0.001	343	Protein	Yes, IHC assay	Higher	(19)
		RFS		0.0026	1.7159	1.2069-2.4396								
EGFR	EGFR	OS	Upper 25%	2e-04	1.9435	1.3703-2.7565	525	Upper *n* = 7 /Lower *n* = 18	OS: *p* < 0.01	25	Protein	Yes, IHC assay	Higher	(20, 21)
		RFS		0.0434	1.4379	1.0108-2.0453								
IDH1	IDH1	OS	Upper 50%	0.0024	1.7174	1.2118-2.4338	525	WT *n* = 108 /MT *n* = 310	OS: *p* = 0.015	418	DNA pyrosequencing		Higher	(22)
		RFS		0.0501	1.3913	0.9999-1.9359								
VEGFA	VEGFA	OS	Upper 25%	<0.0001	2.5754	1.8074-3.6696	525	Upper *n* = 39 /Lower *n* = 35	OS: *p* = 0.002	74	Protein	Yes, IHC assay	Higher	(23, 24)
		RFS		<0.0001	2.1336	1.4857-3.0641								
		PFS	Upper 25%	0.0296	1.604	1.0478-2.4555	195							
NES	nestin	OS	Upper 25%	0.0178	1.5426	1.0779-2.2076	525	Upper *n* = 25 /Lower *n* = 25	OS: *p* = 0.0004	50	Protein	Yes, IHC assay	Higher	(25–27)
		RFS		0.02	1.5177	1.0679-2.1569								
BIRC5	survivin	OS	Upper 30%	<0.0001	2.5472	1.8055-3.5937	525	Upper *n* = 13 /Lower *n* = 8	OS: *p* = 0.007	21	Protein	Yes, IHC assay	Higher	(28)
		RFS		<0.0001	1.9996	1.437-2.7824								
		PFS	Upper 25%	0.0156	1.6915	1.1047-2.5899	195							
PCNA	PCNA	OS	Upper 25%	<0.0001	2.7723	1.9575-3.9263	525		OS: *p* = 0.0009	85	Protein	Yes, IHC assay	Higher	(29)
		RFS		6e-04	1.8521	1.3048-2.629								
MKI67	Ki-67	OS	Upper 30%	<0.0001	2.4146	1.7159-3.3978	525	Upper *n* = 128 /Lower *n* = 52	OS: *p* = 0.047	180	Protein	Yes, IHC assay	Higher	(30–32)
		RFS		<0.0001	2.1909	1.5759-3.0458								
CDKN1B	p27	OS	Lower 50%	0.0073	1.6099	1.137-2.2793	525	Upper *n* = 30 /Lower *n* = 28	OS: *p* = 0.007	77	Protein	Yes, IHC assay	Lower	(21, 33, 34)
		RFS		0.0266	1.455	1.0444-2.027								

*WT, wild type; MT, mutation*.

### Discovery of Novel Potential Prognostic Biomarkers in OSlgg

**Figure 6:**
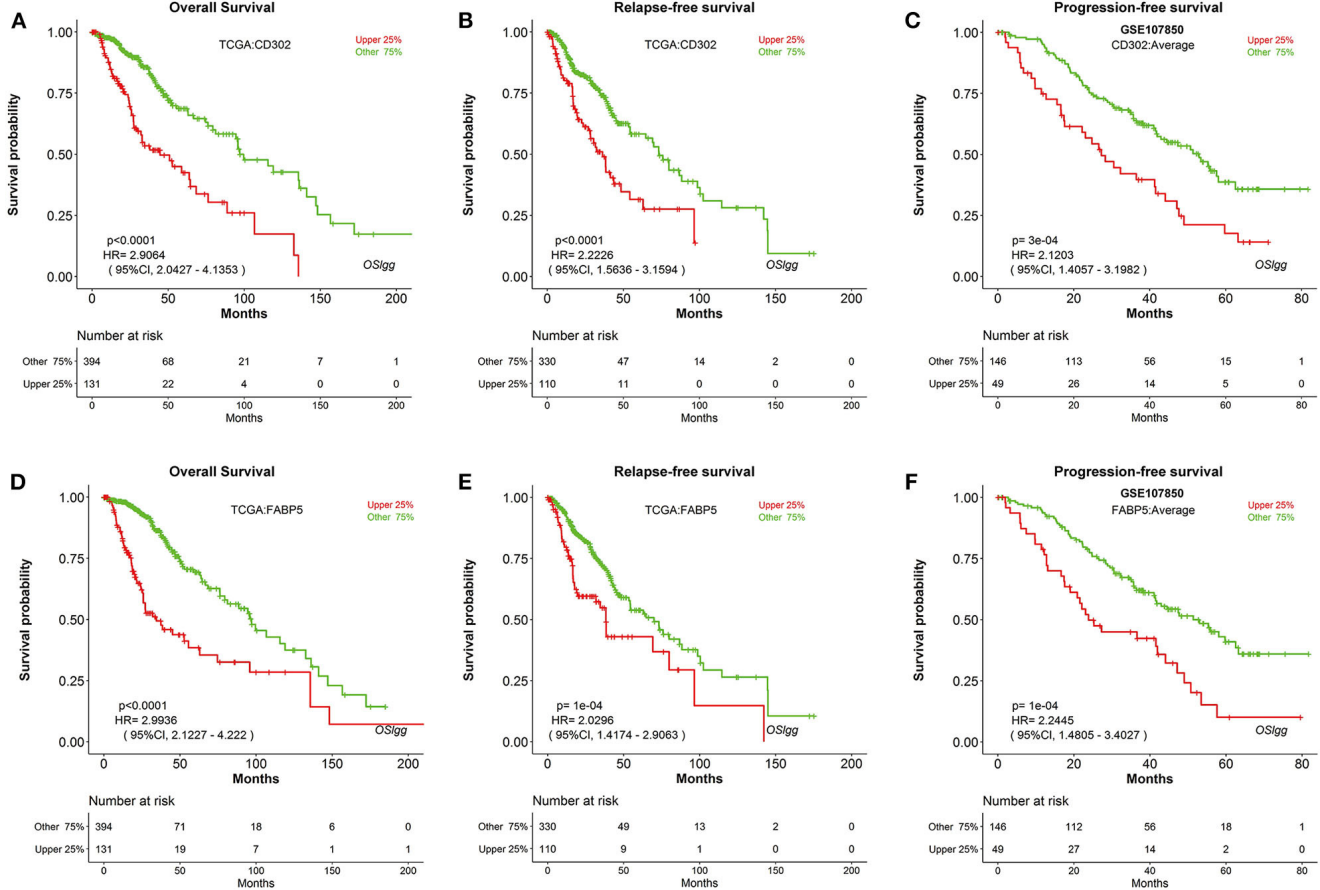
Identification of potential prognostic biomarkers in OSlgg. Kaplan-Meier plots for low (green) and high (red) *CD302* **(A–C)** or *FABP5*-expression **(D–F)** in TCGA cohort **(A,B,D,E)** and GSE107850 cohort **(C,F)**. **(A,D)** OS, Overall survival; **(B,E)** RFS, Relapse-free survival; **(C,F)** PFS, Progression-free survival. *p*-value, confidence interval (95%CI) and number at risk are as shown. The y-axis represents survival rate and the x-axis represents survival time (months).

In order to discover novel risk predictors for LGG, we analyzed the prognostic abilities of all known human genes using Cox regression. As a result, two genes were identified as potential biomarkers, including *CD302* and *FABP5*, which were both significantly associated with survival (OS, RFS and PFS) in OSlgg (Figure 6 and Table 3). Moreover, we found that patients with elevated *CD302* or *FABP5* expression exhibited worse survival in both TCGA (OS and RFS) and GSE107850 (PFS) datasets, while the lower expression patients presented better survival (Figure 6 and Table 3), indicating that both *CD302* and *FABP5* could predict the adverse outcome as unfavorable predictors.

**Table 3:** Identification of potential LGG prognostic biomarkers in OSlgg.

**Gene symbol**	**In OSlgg**					**Case number**
	**Cut-off**	**Survival terms**	***p* value**	**HR**	**95%CI**	
CD302	upper 25%	OS	<0.0001	2.9064	2.0427-4.1353	525
		RFS	<0.0001	2.2226	1.5636-3.1594	525
		PFS	3e-04	2.1203	1.4057-3.1982	195
FABP5	upper 25%	OS	<0.0001	2.9936	2.1227-4.222	525
		RFS	1e-04	2.0296	1.4174-2.9063	525
		PFS	1e-04	2.2445	1.4805-3.4027	195

**Figure 7:**
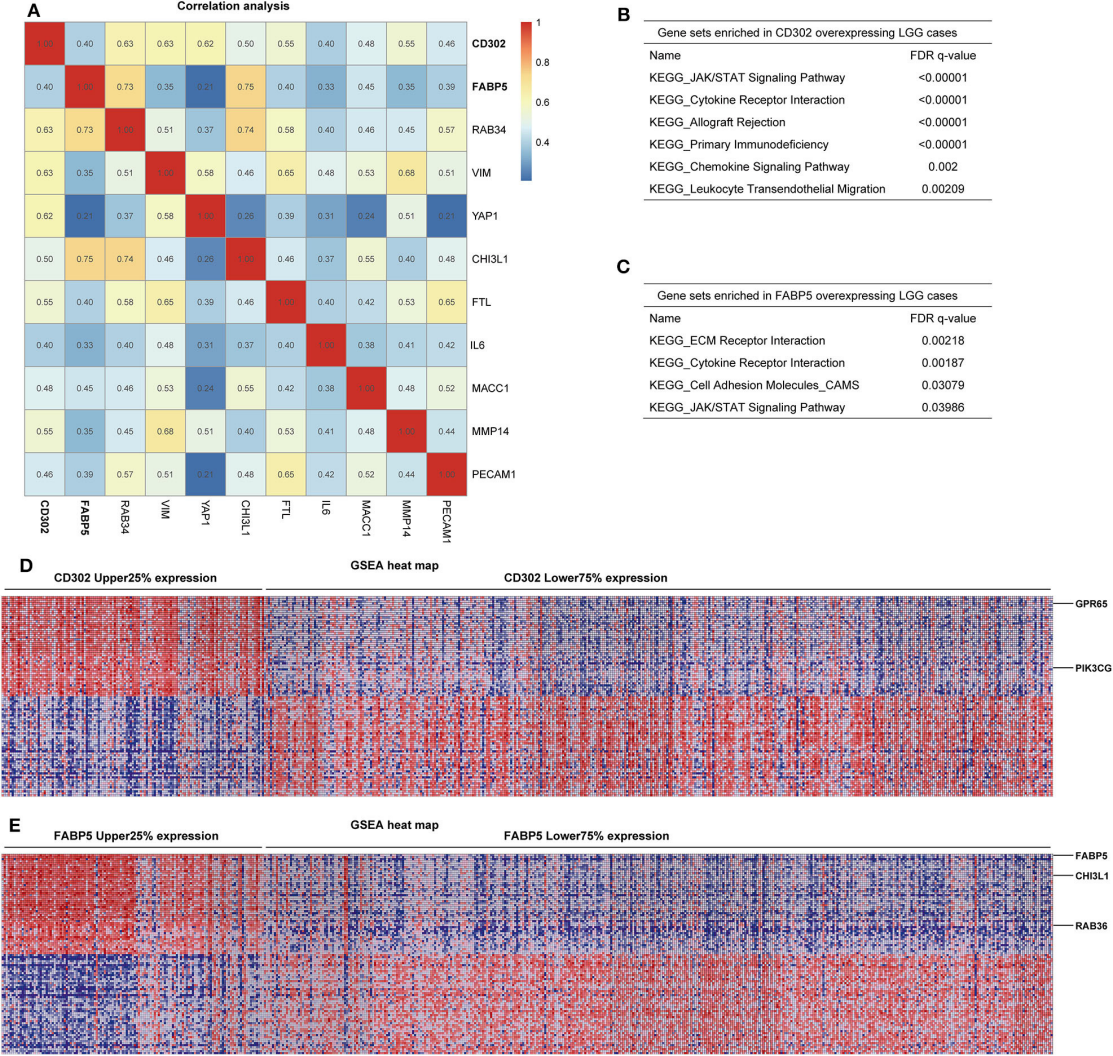
Correlation and GSEA analysis of *CD302* and *FABP5*. **(A)** Correlation analysis between *CD302* or *FABP5* and the previously reported prognostic biomarkers by Spearman's rank correlation test with correlation coefficient marked on the matrix plot. **(B–E)** GSEA analysis of tumors with high versus low expression of *CD302* and *FABP5*. LGG patients were split into two subgroups according to *CD302* or *FABP5* expression, named as CD302 or FABP5 Upper 25% expression and Lower 75% expression. **(B,C)** Gene sets enriched in *CD302* and *FABP5* overexpressing LGG cases, respectively. **(D,E)** GSEA heat maps for differential expression genes enriched in *CD302* and *FABP5* overexpressing LGG cases, respectively.

To determine whether the prognostic significances of *CD302* and *FABP5* are caused by correlation with the previously reported prognostic genes, the correlation analysis between *CD302/FABP5* and the 86 reported prognostic biomarkers were performed, and showed that *CD302/FABP5* were positively correlated with 6 reported prognostic genes, including *RAB34, CHI3L1, VIM, YAP1, FTL*, and *MMP14* (Figure 7A). Among these, *RAB34* is positively associated with both *CD302* and *FABP5, CHI3L1* is positively associated with *FABP5*, and the remaining four genes are all positively correlated with *CD302* (Figure 7A). The GSEA analysis of LGG cases showed that those cases with high *CD302* expression enriched gene sets involved in JAK/STAT signaling pathway, cytokine receptor interaction, and primary immunodeficiency (Figure 7B). And the same analysis found that LGG cases with higher *FABP5* expression enriched gene sets including ECM receptor interaction, cytokine receptor interaction and JAK/STAT signaling pathway (Figure 7C). Moreover, LGG with *CD302* overexpression presented *GPR65* and *PIK3CG* up-regulation, while *CHI3L1* and *RAB36* were up-regulated in tumors with *FABP5* overexpression (Figures 7D,E). In addition, we found that *GPR65, PIK3CG*, and *RAB36* have prognostic abilities in LGG, the elevated expression of which were significantly associated with worse survival of LGG patients (Table S2 and Figure S3). As Figure S4 showed, there is no significant difference of the copy numbers between *CD302* or *FABP5* higher and lower expression groups, respectively, indicating the prognostic significance of *CD302* and *FABP5* is not caused by genomic copy number changes.

### Independent Prognostic and Clinical Significance of CD302 and FABP5

To further investigate the relationship between *CD302*/*FABP5* and clinical factors, we analyzed the expression differences of *CD302*/*FABP5* between LGG subgroups with distinct clinical features, the results showed that LGG patients suffered from histologic grade 3 and progressive disease had significant higher expression of *CD302*/*FABP5*, respectively (Figure S5). In addition, as shown in Table 4, the expression of *CD302*/*FABP5* was significantly associated with histologic grade and primary therapy outcome. The higher *CD302* and *FABP5* expression subgroup presented a significantly higher ratio of patients in histologic grade 3 (91/40 vs. 178/215, *p* < 0.001 and 85/46 vs. 184/209, *p* < 0.001) compared to the lower *CD302* and *FABP5* subgroup, respectively (Table 4). The following multivariate analysis confirmed that the elevated *CD302*/*FABP5* expression is an independent prognostic indicator of LGG survival (HR: 1.842, 95% CI: 1.232-2.754, *p* = 0.003, and HR: 2.187, 95% CI: 1.488-3.214, *p* < 0.001), respectively (Table 5).

**Table 4:** The association of CD302 or FABP5 expression with clinical features in LGG patients.

**Variables**	**No. of patient**	**CD302 expression**		**χ2**	***p* value**	**FABP5 expression**		**χ2**	***p* value**
		**Upper** **25%**	**Lower** **75%**			**Upper** **25%**	**Lower** **75%**		
Histologic grade				22.981	< 0.001			12.836	< 0.001
G2	255	40	215			46	209		
G3	269	91	178			85	184		
Unknown	1								
Therapy outcome				29.313	< 0.001			28.983	< 0.001
Complete response	133	17	116			17	116		
Partial response	65	11	54			10	55		
Progressive disease	114	47	67			46	68		
Stable disease	137	34	103			32	105		
Unknown	76								

**Table 5:** Univariate and multivariate analysis of factors associated with LGG survival.

**Subgroup**	**Univariate Analysis**		**Multivariate Analysis**	
	**Hazard ratio (95% CI)**	***p* value**	**Hazard ratio (95% CI)**	***p* value**
All patients (*N* = 525)				
Histologic type	0.757 (0.621–0.922)	0.006	0.903 (0.725–1.125)	0.363
Histologic grade (*N* = 524)	3.354 (2.298–4.895)	<0.001	2.387 (1.577–3.612)	<0.001
Primary therapy outcome (*N* = 449)	1.527 (1.267–1.839)	<0.001	1.461 (1.201–1.777)	<0.001
CD302 expression	2.899 (2.038–4.124)	<0.001	1.842 (1.232–2.754)	0.003
FABP5 expression	2.984 (2.116–4.208)	<0.001	2.187 (1.488–3.214)	<0.001

**Figure 8:**
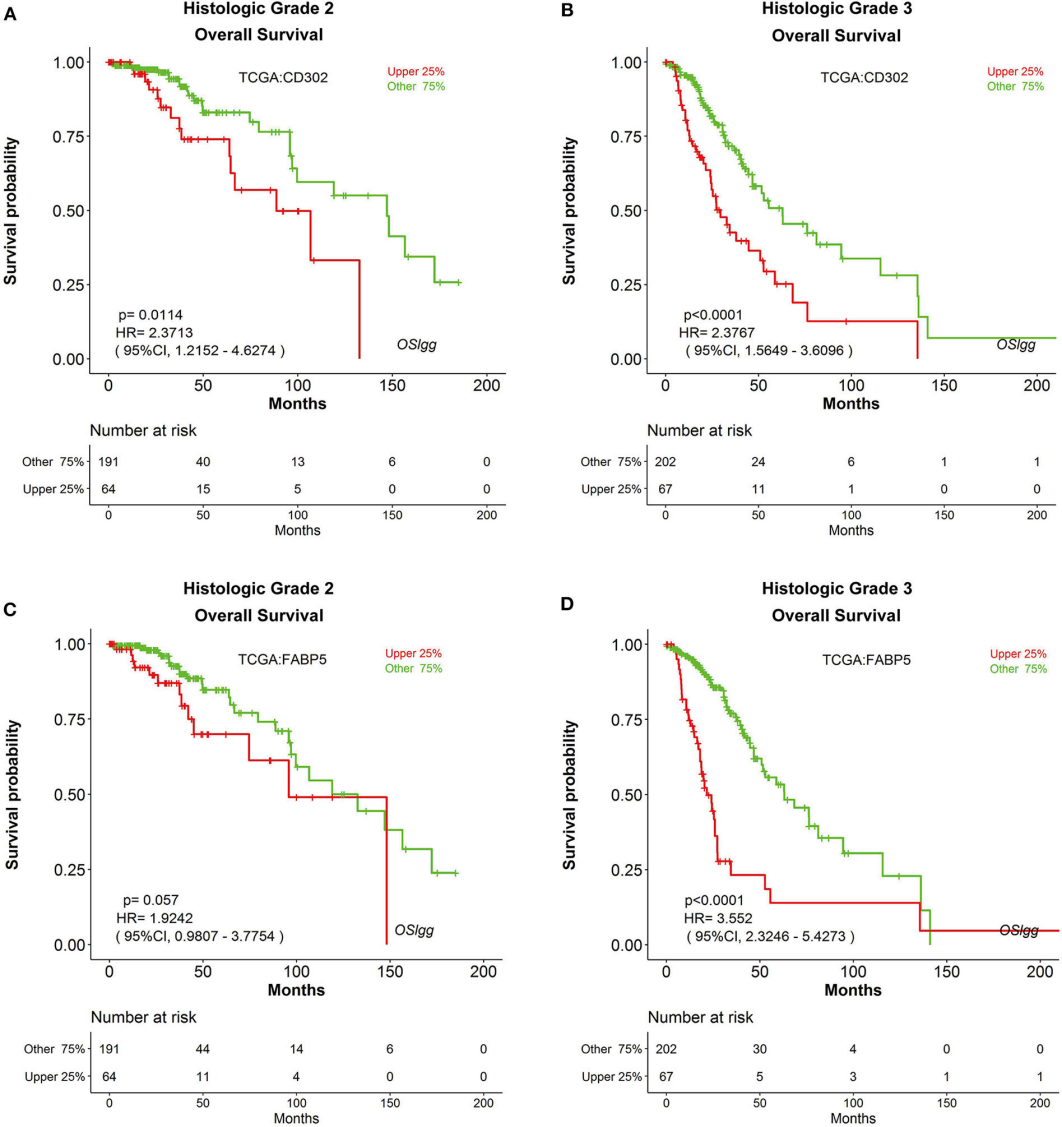
The prognostic abilities of *CD302* and *FABP5* in terms of histologic grade. Kaplan-Meier plots for *CD302* in histologic grade 2 **(A)** and 3 **(B)**, and for *FABP5* in histologic grade 2 **(C)** and 3 **(D)**, respectively. *p*-value, confidence interval (95%CI) and number at risk are as shown. The y-axis represents survival rate and the x-axis represents survival time (months).

**Figure 9:**
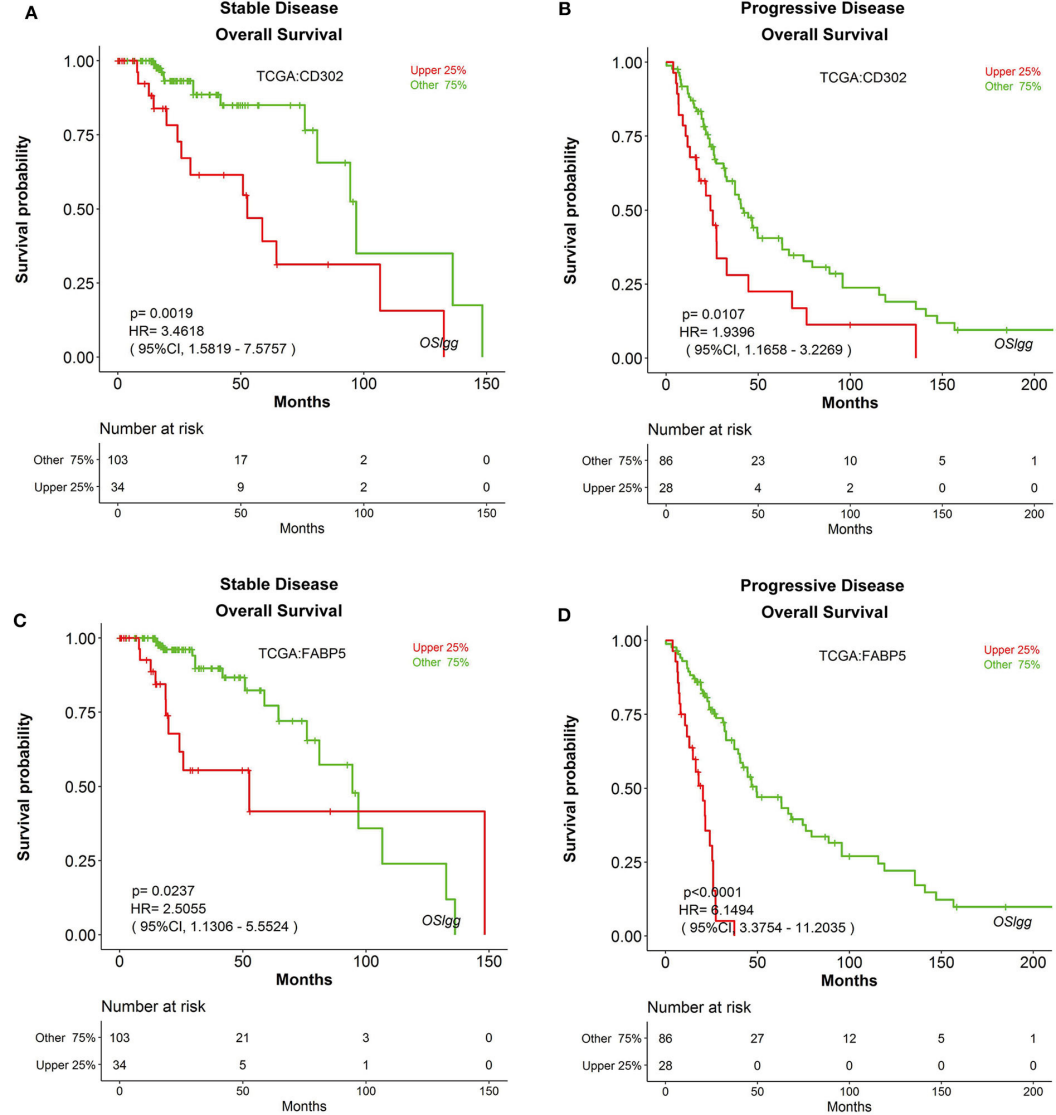
The prognostic abilities of *CD302* and *FABP5* in terms of primary therapy outcome. Kaplan-Meier plots for *CD302* in stable **(A)** and progressive **(B)** disease, and for *FABP5* in stable **(C)** and progressive **(D)** disease, respectively. *p*-value, confidence interval (95%CI) and number at risk are as shown. The y-axis represents survival rate and the x-axis represents survival time (months).

Furthermore, we also found that the prognostic abilities of *CD302* and *FABP5* were independent of the critical clinical features of LGG patients, including histologic grade, therapy and primary therapy outcome (Figures 8, 9, Figures S6, S7). In detail, patients with *CD302*/*FABP5* overexpression exhibited worse survival in both histologic grade 2 and 3 (Figure 8), both stable and progressive disease (Figure 9), and both radiotherapy and TMZ (temozolomide) therapy (Figures S6, S7), while no significant prognostic significance of *CD302/FABP5* observed in patients with complete and partial response.

## Discussion

Gliomas are graded as I to IV according to the histology and clinical criteria. Grade II and III glioma are designated as low-grade glioma (LGG) (1–4). Although LGG accounts for a minority of gliomas, it is the major cause of mortality for young adults (14). Although the survival outcomes for patients diagnosed with LGG are better than those for high-grade gliomas, LGG almost universally advances to high-grade glioma (5, 8). Surgical resection is the major treatment for LGG. However, even under gross total resection (GTR), the survival rates of LGG patients are still low, having the risk of tumor progression (9). Some low-risk patients exhibit tumor progression-free without intervention, while others with high-risk suffer from the progressive disease, for which intervention treatment may be given after being diagnosed (6). As the patients suffering from LGG have distinct clinical performances, it is necessary to classify patients into subgroups with different risks to guide following treatments.

In this study, we developed a web server OSlgg, by which users could evaluate the prognostic value of genes of interest even for users with limited bioinformatics skills. To determine the reliability of OSlgg, we have verified the prognostic roles of 86 previously reported LGG prognostic biomarkers including IDH1, BIRC5, CDKN1B, PCNA, and MKI67. Furthermore, we have identified two novel potential prognostic biomarkers for LGG patients, including CD302 and FABP5. As C-type lectin receptor, CD302 has roles in cell immune and migration (35, 36), and acts as a prognostic biomarker in myeloma (37), is also a potential therapeutic target for acute myeloid leukemia (38). In addition, CD302 had been identified as a biomarker to categorize the metastases of neuroendocrine tumors (NET) (39), and it is reported to be overexpressed in high grade NET (40). Fatty acid-binding protein 5 (FABP5) is involved in fatty acid transport, and acts as a prognostic biomarker in cervical cancer, triple-negative breast cancer and clear cell renal cell carcinoma (41–43). In addition, FABP5 was found to be expressed in 9 of 23 gliomas with moderate to strong cytoplasmic staining in Human Protein Atlas (HPA) database, and was reported to be expressed in grade II (19/30) and III (22/31) astrocytoma (a histologic subtype of glioma) (44). The prognostic abilities of CD302 and FABP5 have not been reported in LGG yet. In our server, the cox regression analysis reveals that CD302 and FABP5 are significantly correlated with survival outcomes of LGG patients, patients with lower expression of CD302 and FABP5 have improved outcomes compared to patients with higher expression of these genes, and we found that the elevated *CD302*/*FABP5* expression was significantly associated with higher histologic grade and worse therapeutic outcome, in the meanwhile, we found that CD302 and FABP5 were independent prognostic indicators of LGG.

Additional correlation analysis showed that *CD302* and *FABP5* were significantly correlated with 6 of 86 reported unfavorable prognostic biomarkers including *RAB34, CHI3L1, VIM, YAP1, FTL*, and *MMP14*, which predicted adverse outcome (45–50). These six *CD302/FABP5* correlated genes were reported to be involved in tumor cell proliferation, migration, invasion and EMT (46–53). GSEA results showed LGG tumors with high expression of *CD302* or *FABP5* enriched JAK/STAT and ECM receptor interaction signaling pathway, which are reported to be involved in tumorigenesis and could promote tumor progression (54, 55). Moreover, LGG tumors with *CD302* or *FABP5* overexpression highly expressed some oncogenes, including *GPR65, PIK3CG, CHI3L1*, and *RAB36*, which were reported to promote tumor growth and metastasis (56–60). Taken together, our results highlight the clinical significance of *CD302* and *FABP5* in LGG, the expression of which may have a close association with tumorigenesis and malignant progression of LGG. Further assays for biological functions of these genes may offer opportunities for targeted therapies in LGG.

The limitation of OSlgg is that currently only 720 LGG cases are available in our server. Once new datasets with profiling and clinical follow-up data become available, we will update OSlgg to expand the dataset and enhance the performance.

In summary, we developed a prognosis analysis web server OSlgg, which provides a platform for researchers and clinicians to evaluate the prognostic values of genes of interest, and may offer opportunities to facilitate the development of novel targeted strategies for LGG.

## Data Availability Statement

Publicly available datasets were analyzed in this study. This data can be found here: TCGA database (https://www.cancer.gov/about-nci/organization/ccg/research/structural-genomics/tcga) and GEO database (https://www.ncbi.nlm.nih.gov/gds/?term=).

## Author Contributions

YA, QW, and XG developed the server, performed the evaluation of novel prognostic biomarkers, and drafted the paper. LZ, FS, GZ, and HD performed the validation of previous reported biomarkers. HL, YL, and YP collected LGG datasets. WZ, SJ, and YW contributed to data analysis and paper revision. All authors approved the final manuscript.

## Conflict of Interest

The authors declare that the research was conducted in the absence of any commercial or financial relationships that could be construed as a potential conflict of interest.

## Supplementary Material

Figure S1 Verification of previously reported prognostic biomarkers in OSlgg. Kaplan-Meier plots for **(A)** *CDH2*, **(B)** *EGFR*, **(C)** *IDH1*, **(D,J)** *VEGFA*, **(E)** *NES*, **(F,K)** *BIRC5*, **(G)** *PCNA*, **(H)** *MKI67* and **(I)** *CDKN1B* in terms of RFS and PFS. **(A–I)** RFS, Relapse-free survival, in TCGA cohort; **(J,K)** PFS, Progression-free survival, in GSE107850 cohort.

Figure S2 Kaplan-Meier plots for housekeeping genes as negative controls. **(A)** *TUBB1*, **(B)** *TUBB3* and **(C)** *ACTA1*, were presented as negative control genes of Figure 5.

Figure S3 Prognostic analysis of reported oncogenes up-regulated in *CD302*/*FABP5* overexpression cohort. Kaplan-Meier plots for **(A)** *GPR65*, **(B)** *PIK3CG*, **(C)** *CHI3L*1 and **(D)** *RAB36*. *p*-value, confidence interval (95%CI) and number at risk are as shown. The y-axis represents survival rate and the x-axis represents survival time (months).

Figure S4 Analysis of the relationship between mRNA expression and copy number variation of *CD302* **(A)** and *FABP5* **(B)** in 508 LGG patients.

Figure S5 Comparison of *CD302/FABP5* expression differences in distinct clinical features. **(A,B)** histologic grade, **(C,D)** primary therapy outcome. TPM, Transcripts Per Million.

Figure S6 The prognostic abilities of *CD302* and *FABP5* in terms of treatment in TCGA cohort. **(A)** Kaplan-Meier plots for radiotherapy treatment in all tumors. **(B,C)** Kaplan-Meier plots for *CD302* high vs. low expression in tumors with and without radiotherapy, respectively. **(D,E)** Kaplan-Meier plots for *FABP5* high vs. low expression in tumors with and without radiotherapy, respectively. *p*-value is as shown. The y-axis represents survival rate and the x-axis represents survival time (months).

Figure S7 The prognostic abilities of *CD302* and *FABP5* in terms of treatment in GSE107850 cohort. **(A,B)** Kaplan-Meier plots for *CD302* in radiotherapy and TMZ (temozolomide) therapy, respectively. **(C,D)** Kaplan-Meier plots for *FABP5* in radiotherapy and TMZ (temozolomide) therapy, respectively. *p*-value, confidence interval (95%CI) and number at risk are as shown. The y-axis represents survival rate and the x-axis represents survival time (months).

Table S1 Clinicopathologic Characteristics of LGG patients.

Table S2 Verification of previous published predictors for LGG survival in OSlgg.
