# Supplementary figures and images for: OSlgg: An Online Prognostic Biomarker Analysis Tool for Low-Grade Glioma
Source: Front Oncol. 2020 Jul 7;10:1097. doi: 10.3389/fonc.2020.01097 (PMC7381343; doi:10.3389/fonc.2020.01097)

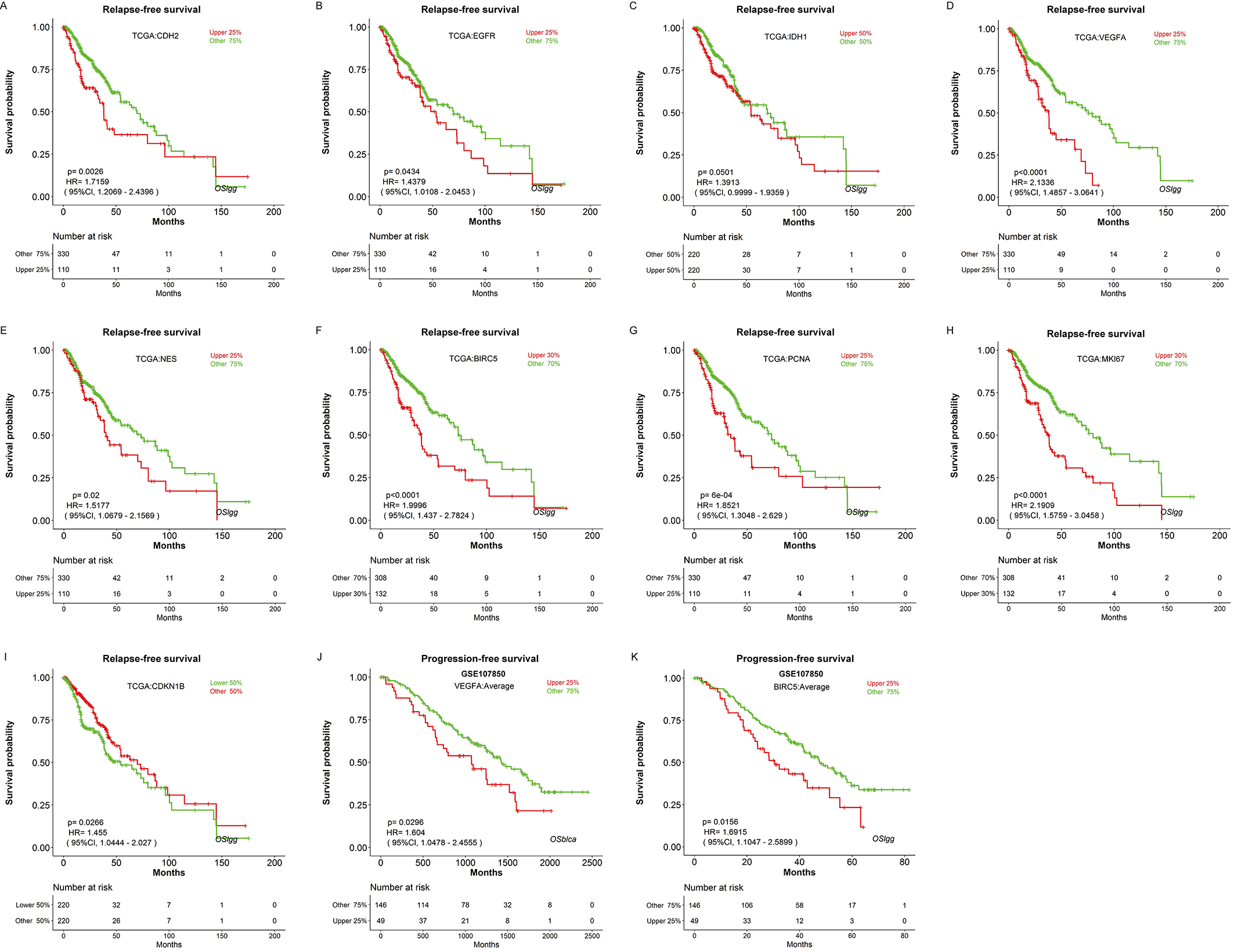

Supplement: Figure S1 — Verification of previously reported prognostic biomarkers in OSlgg. Kaplan-Meier plots for (A) CDH2, (B) EGFR, (C) IDH1, (D,J) VEGFA, (E) NES, (F,K) BIRC5, (G) PCNA, (H) MKI67 and (I) CDKN1B in terms of RFS and PFS. (A–I) RFS, Relapse-free survival, in TCGA cohort; (J,K) PFS, Progression-free survival, in GSE107850 cohort. [file Image_1.TIF]

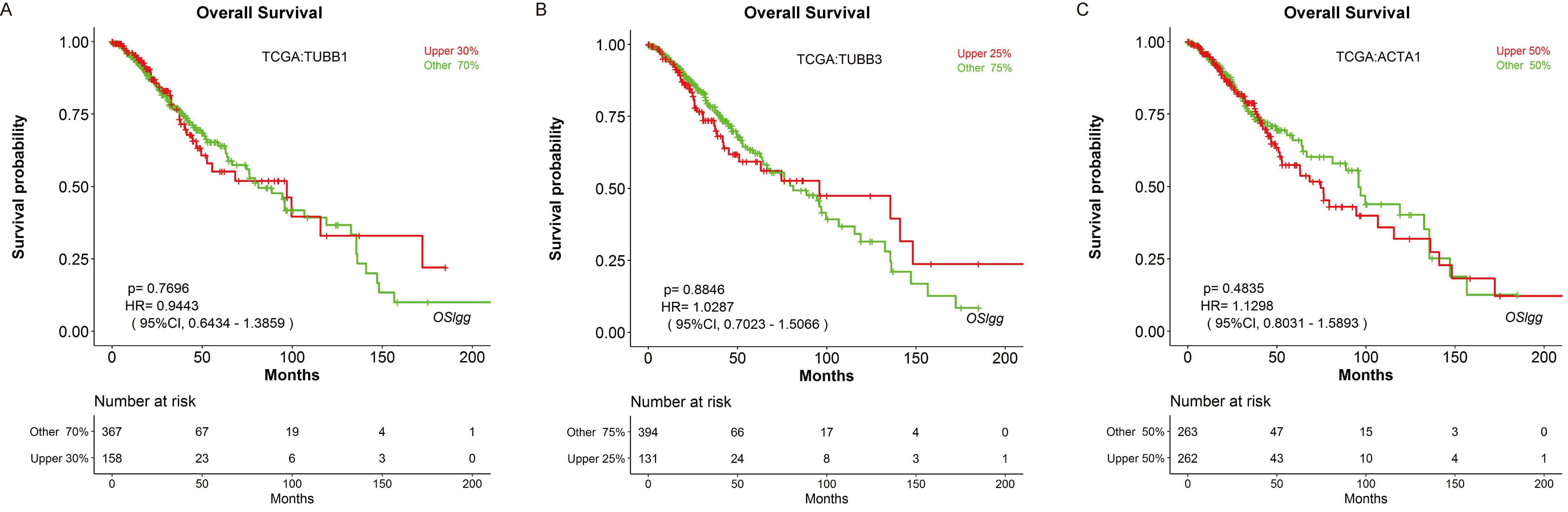

Supplement: Figure S2 — Kaplan-Meier plots for housekeeping genes as negative controls. (A) TUBB1, (B) TUBB3 and (C) ACTA1, were presented as negative control genes of Figure 5. [file Image_2.TIF]

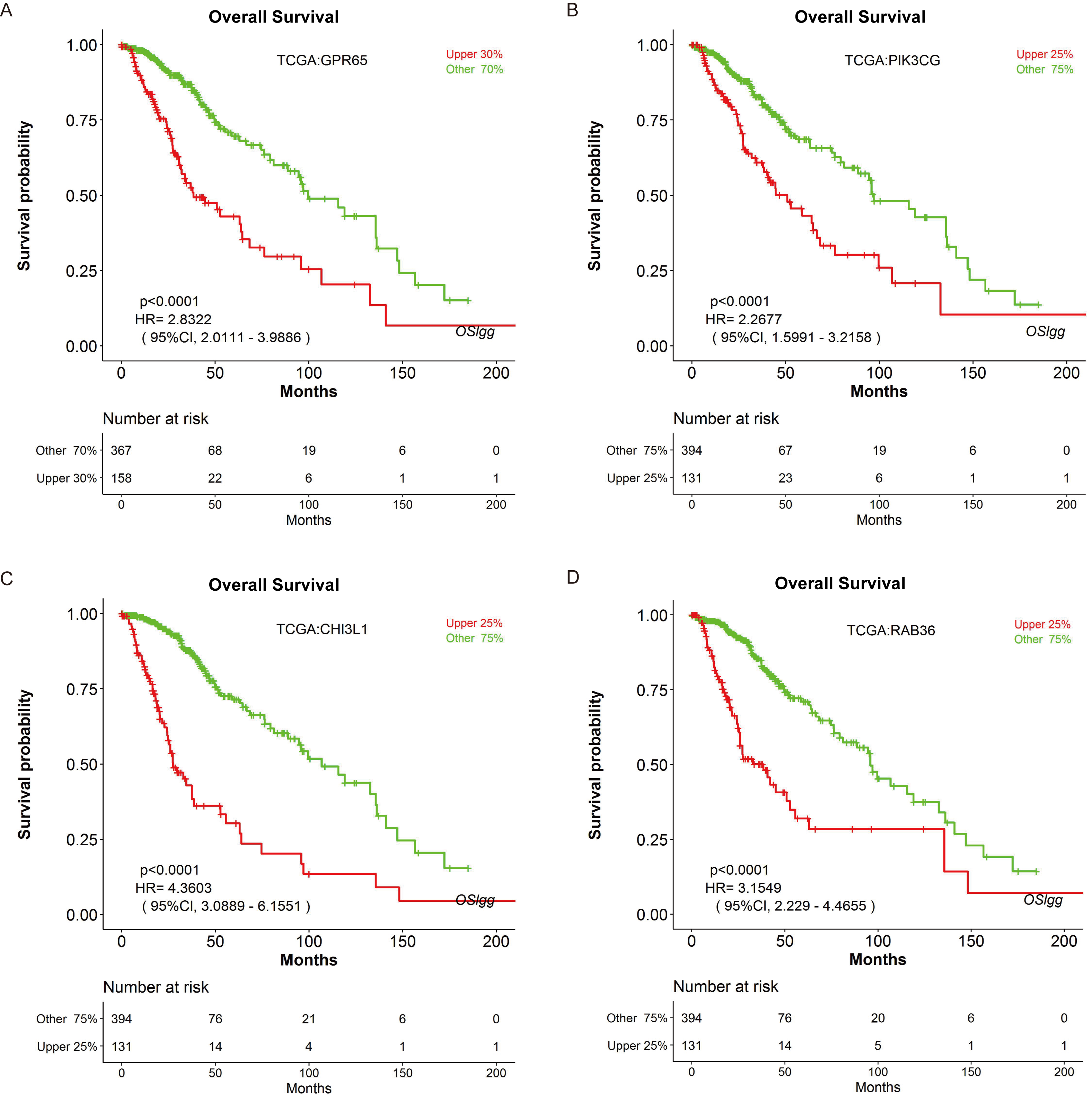

Supplement: Figure S3 — Prognostic analysis of reported oncogenes up-regulated in CD302/FABP5 overexpression cohort. Kaplan-Meier plots for (A) GPR65, (B) PIK3CG, (C) CHI3L1 and (D) RAB36. p-value, confidence interval (95%CI) and number at risk are as shown. The y-axis represents survival rate and the x-axis represents survival time (months). [file Image_3.TIF]

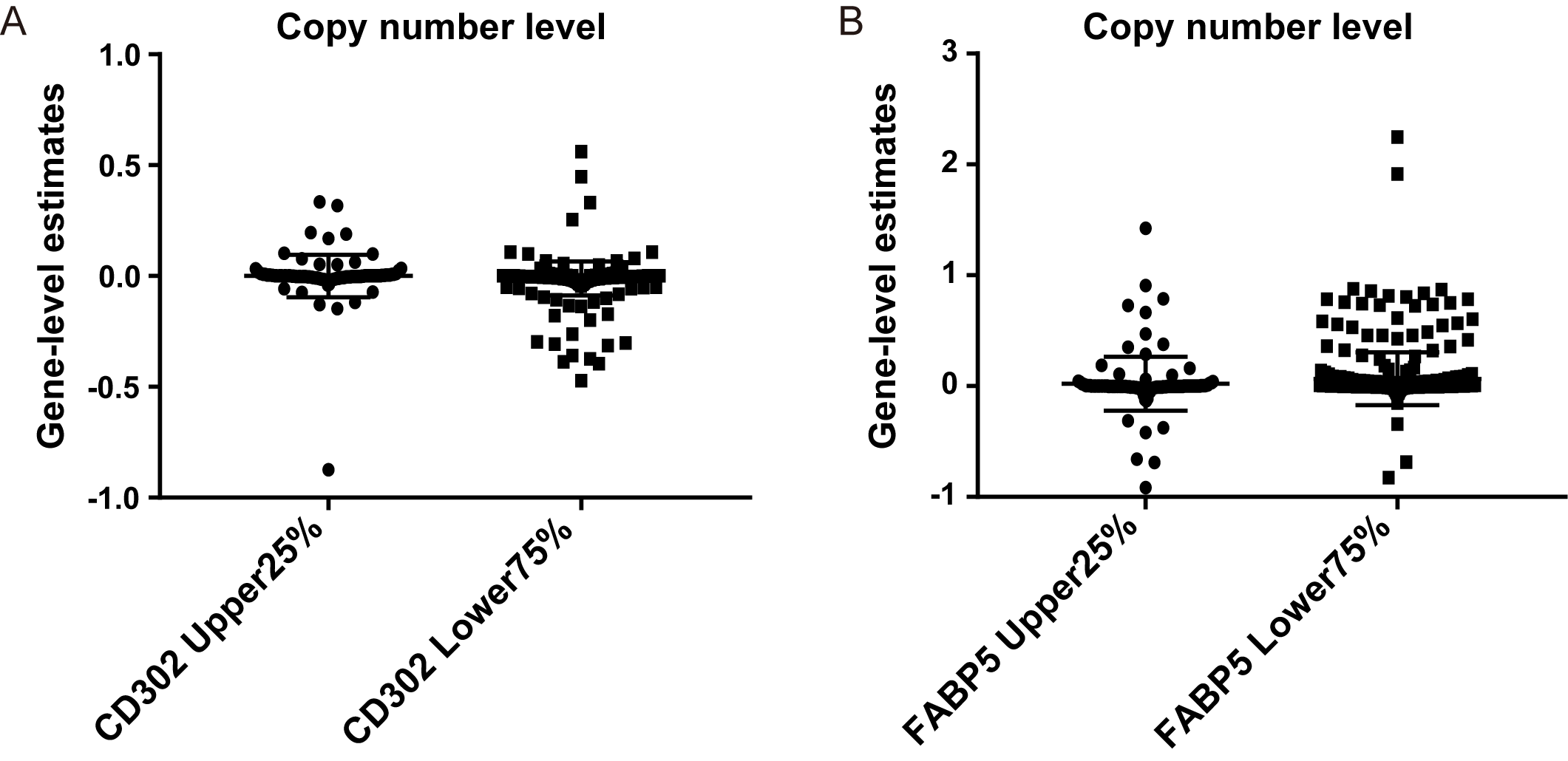

Supplement: Figure S4 — Analysis of the relationship between mRNA expression and copy number variation of CD302 (A) and FABP5 (B) in 508 LGG patients. [file Image_4.TIF]

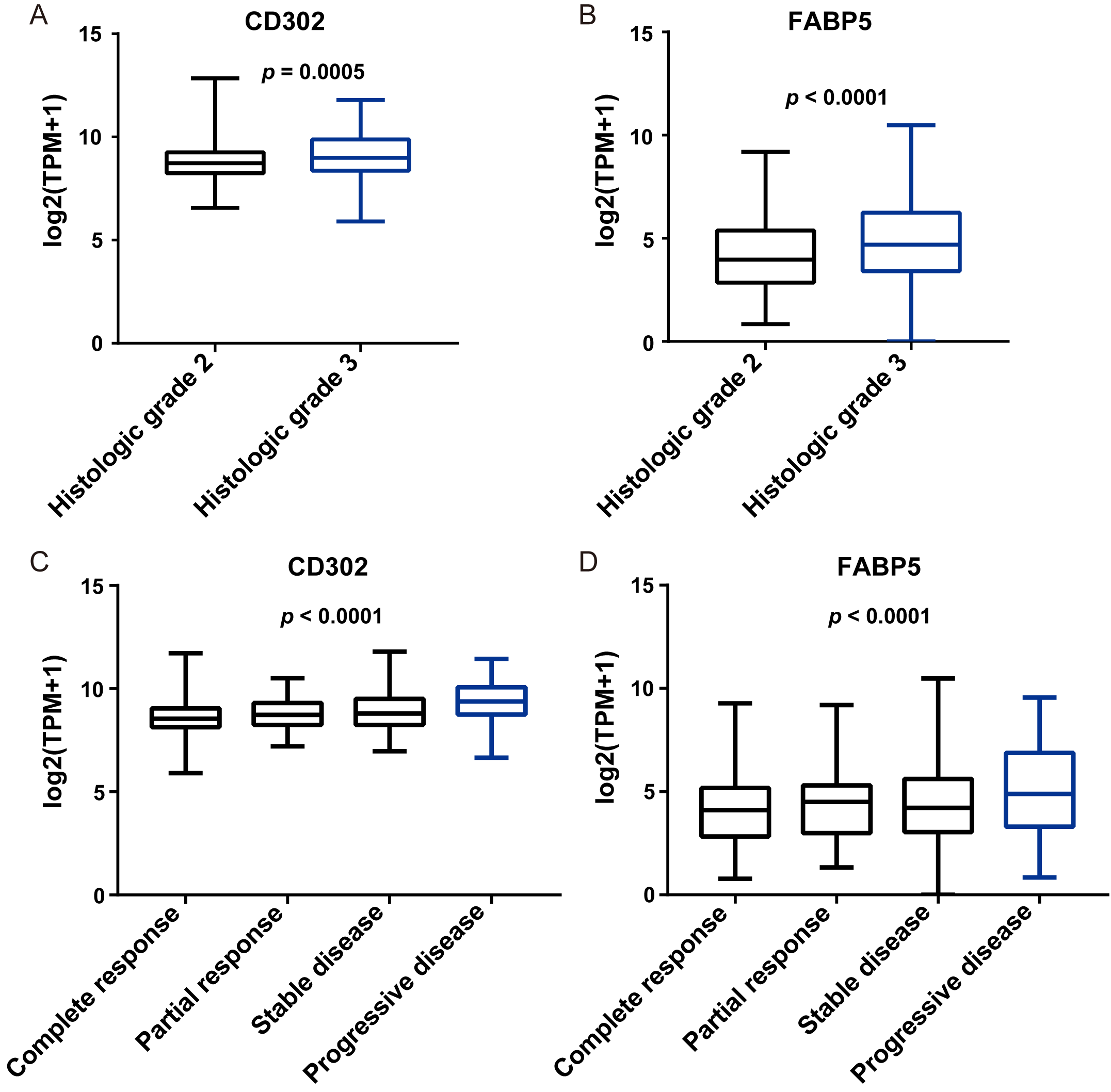

Supplement: Figure S5 — Comparison of CD302/FABP5 expression differences in distinct clinical features. (A,B) histologic grade, (C,D) primary therapy outcome. TPM, Transcripts Per Million. [file Image_5.TIF]

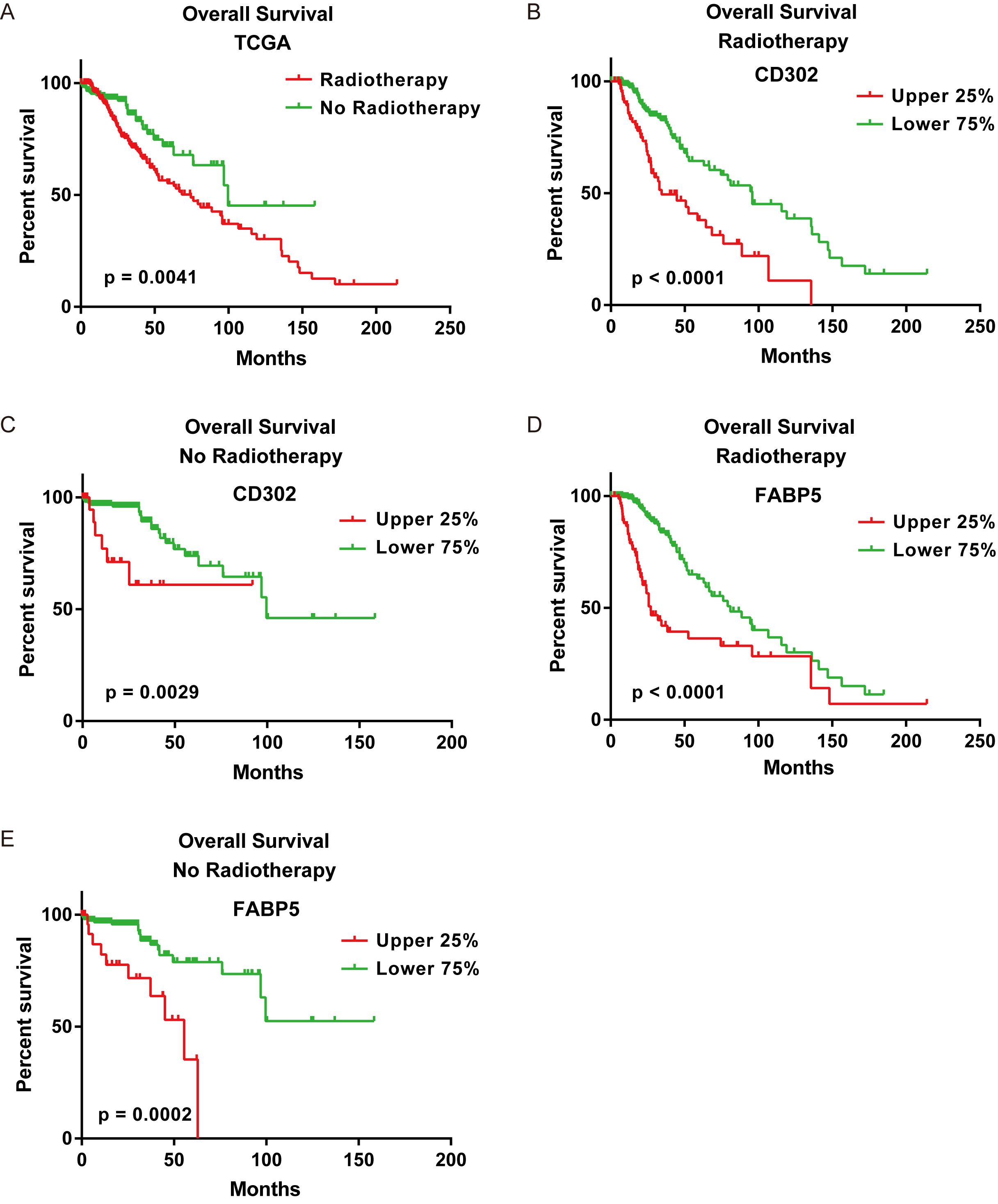

Supplement: Figure S6 — The prognostic abilities of CD302 and FABP5 in terms of treatment in TCGA cohort. (A) Kaplan-Meier plots for radiotherapy treatment in all tumors. (B,C) Kaplan-Meier plots for CD302 high vs. low expression in tumors with and without radiotherapy, respectively. (D,E) Kaplan-Meier plots for FABP5 high vs. low expression in tumors with and without radiotherapy, respectively. p-value is as shown. The y-axis represents survival rate and the x-axis represents survival time (months). [file Image_6.TIF]

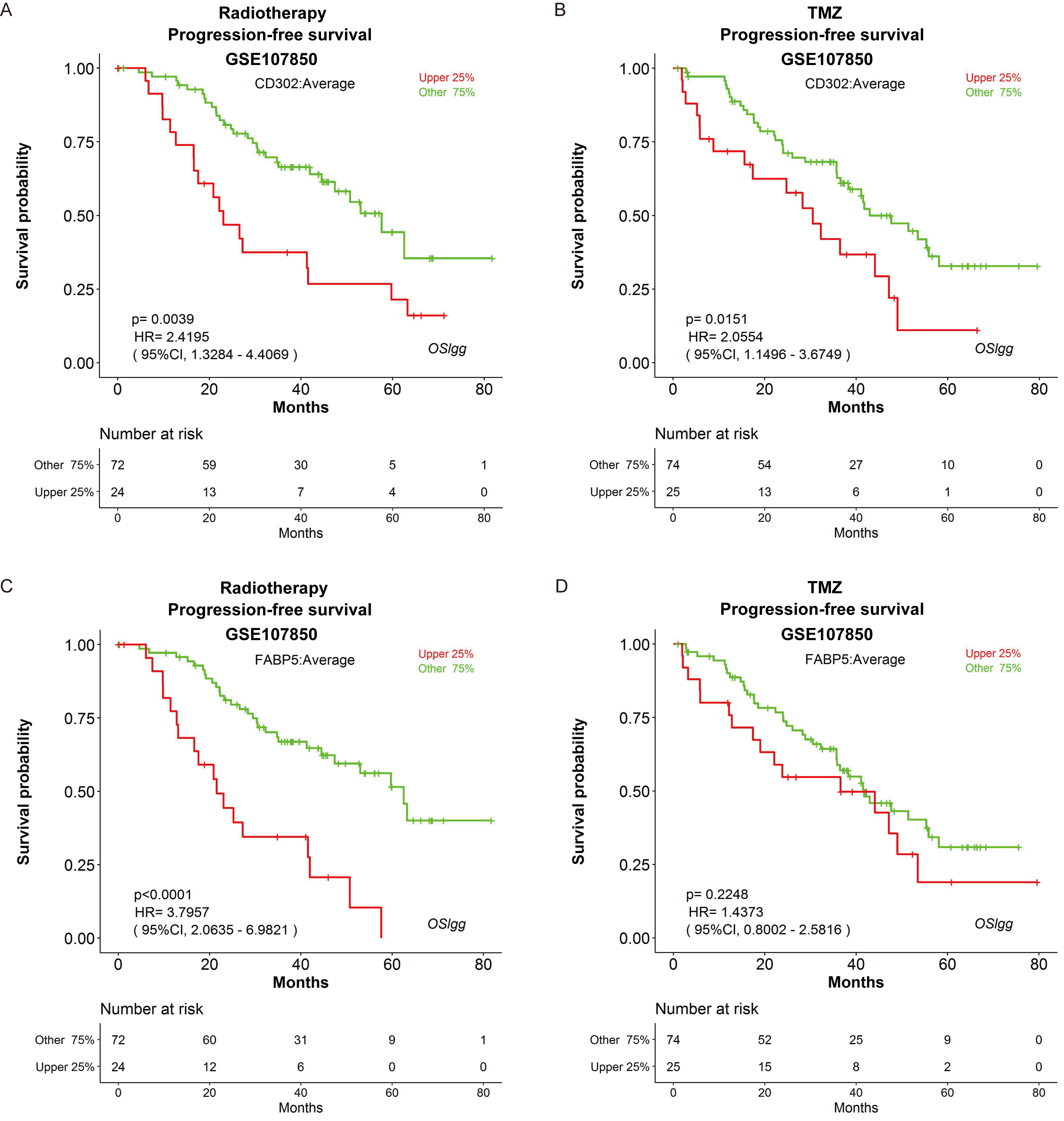

Supplement: Figure S7 — The prognostic abilities of CD302 and FABP5 in terms of treatment in GSE107850 cohort. (A,B) Kaplan-Meier plots for CD302 in radiotherapy and TMZ (temozolomide) therapy, respectively. (C,D) Kaplan-Meier plots for FABP5 in radiotherapy and TMZ (temozolomide) therapy, respectively. p-value, confidence interval (95%CI) and number at risk are as shown. The y-axis represents survival rate and the x-axis represents survival time (months). [file Image_7.TIF]
